# Maximal fat oxidation training improves mental health in children with obesity: a 2-month randomized controlled trial

**DOI:** 10.3389/fspor.2026.1759324

**Published:** 2026-02-25

**Authors:** Emna Makni, Monèm Jemni, Mohamed Abdelkader, Mehdi Ben Brahim, Mohamed Elloumi

**Affiliations:** 1 Research Laboratory of Exercise Physiology and Pathophysiology (LR19ES09), Faculty of Medicine Ibn Al-Jazzar, University of Sousse, Sousse, Tunisia; 2 Department of Neurology, Carrick Institute, Cape Canaveral, FL, United States; 3 Faculty of Physical Education, Ningbo University, Ningbo, Zhejiang, China; 4 Centre for Mental Health Research in Association, University of Cambridge, Cambridge, United Kingdom; 5 Department of Statistics and Operations Research, College of Science, King Saud University, Riyadh, Saudi Arabia; 6 Sport Sciences and Diagnostics Research Group, College of Sciences and Humanities, Prince Sultan University, Riyadh, Saudi Arabia

**Keywords:** anxiety, cardiopulmonary fitness, childhood, depression, obesity, self-esteem

## Abstract

**Introduction:**

Obesity impairs physical and mental health; exercise training is a crucial intervention strategy. We examined the effect of an 8-week individualized FATmax training program on mental health in children with obesity.

**Methods:**

Thirty-six school-aged children with obesity (13.1 ± 0.9 years; BMI: 33.4 ± 2.3 kg/m²) were randomized to experimental (EXPG, *n* = 20) or control (CONTG, *n* = 16) groups. EXPG completed 4 × 90-min weekly FATmax sessions for 8 weeks; CONTG maintained usual activity. Anthropometrics, cardiopulmonary fitness (VO₂ peak, FATmax rate), and psychological outcomes (self-esteem, anxiety, depression) were assessed pre- and post-intervention.

**Results:**

EXPG showed significant improvements vs. CONTG: VO₂ peak (+23%), FATmax rate (+66%), and large reductions in central adiposity (all *p* < 0.001). Mental health significantly improved in EXPG (self-esteem +59%, anxiety −12%, depression −28%; all *p* < 0.01), with large effect sizes (*η*_p_² = 0.78–0.94). Changes in mental health strongly correlated with reduced waist circumference (*r* = 0.74–0.89) and enhanced VO₂ peak and FATmax rate (*r* = 0.72–0.91; all *p* < 0.01).

**Discussion:**

A short, supervised, individualized FATmax intervention substantially improves mental health and cardiometabolic health in school-aged children with obesity, plausibly mediated by decreases in central adiposity and improvements in aerobic fitness.

## 1 Introduction

Childhood obesity is a global public health issue (1) caused by genetic, epigenetic, and environmental factors resulting in metabolic, cardiopulmonary, and psychological disorders (2, 3). Several studies found that school-aged children and adolescents with obesity often experience high levels of anxiety and depression, lower self-esteem, and difficulties in emotion regulation, with anxiety being particularly significant and impactful (4, 5). These psychological challenges can negatively impact treatment, making it crucial to screen and treat these conditions (5). Obesity requires a multidisciplinary approach for effective management rather than relying on a single method (6). Blanco et al. (4) found that school-aged children with obesity experience challenges such as poor self-esteem, anxiety, depression, and weight-related bullying, which significantly impair their quality of life. Weight-related teasing was identified as a mediator between body mass index and psychological well-being. These children also commonly experience poor self-esteem, anxiety, and depression. Blanco et al. (4) emphasize the significance of early intervention in identifying and addressing weight-related teasing and psychological issues among children and adolescents with obesity. They state that reducing the burden of obesity-related mental and psychological conditions is equally important as weight reduction (4, 5).

Physical activity has been confirmed as a key strategy for reducing the physical, metabolic, and psychological effects of obesity (6–8). Targeted interventions, particularly when integrated with a holistic approach, significantly improve body composition and fat oxidation outcomes in this population (9, 10). The literature suggests various physical activity modalities including maximal fat oxidation (FATmax) exercise training to counteract obesity's negative effects (9, 11–13). FATmax exercise training enhances body fat burning during moderate-intensity aerobic exercise (9, 11). This strategy is effective in treating chronic diseases, normalizing metabolism, and regulating hormone concentrations in individuals with obesity, as widely adopted by clinicians (9, 11–13). Furthermore, research indicates that the positive effect of this type of training is long-lasting and persists for at least 90 months (14, 15). A recent review suggests that FATmax exercise training is the preferred method for individuals with obesity (9), with higher adherence to exercise training. Emphasizing fat oxidation rates during exercise is more effective for preventing and managing obesity than energy expenditure (15). However, studies on the benefits of training programs on psychological well-being and mental health outcomes in school-aged children with obesity remain limited (8, 16). Yu et al. (8) conducted a school-based intervention combining nutrition education with moderate aerobic games, while Migueles et al. (16) focused on an aerobic and resistance training regimen without emphasizing fat oxidation kinetics. In addition, there is still a gap in research regarding the impact of maximal fat oxidation exercise training on the mental health and well-being of individuals with obesity. Higher levels of cardiorespiratory fitness have been linked to improved mental health in youth, associated with reduced anxiety and depression symptoms, as well as increased self-esteem (17). These associations are mediated by physiological factors such as neurotrophic factors and cerebral blood flow, alongside psychosocial factors like enhanced self-efficacy and body satisfaction (18). We hypothesize that an 8-week FATmax training program will improve mental health, body composition, and cardiopulmonary fitness in school-aged children with obesity compared to controls. The study aims to investigate the effect of an individualized FATmax training program on the mental health profile of school-aged children and adolescents with obesity and its relationship with central obesity and cardiopulmonary markers.

## 2 Materials and methods

### 2.1 Study design

The study used a longitudinal design to assess the impact of a maximum fat oxidation training program on the psychological well-being, mental health, central fat, and cardiopulmonary biomarkers of school-aged children with obesity compared to an age- and body composition-matched control group without intervention.

**Figure 1:**
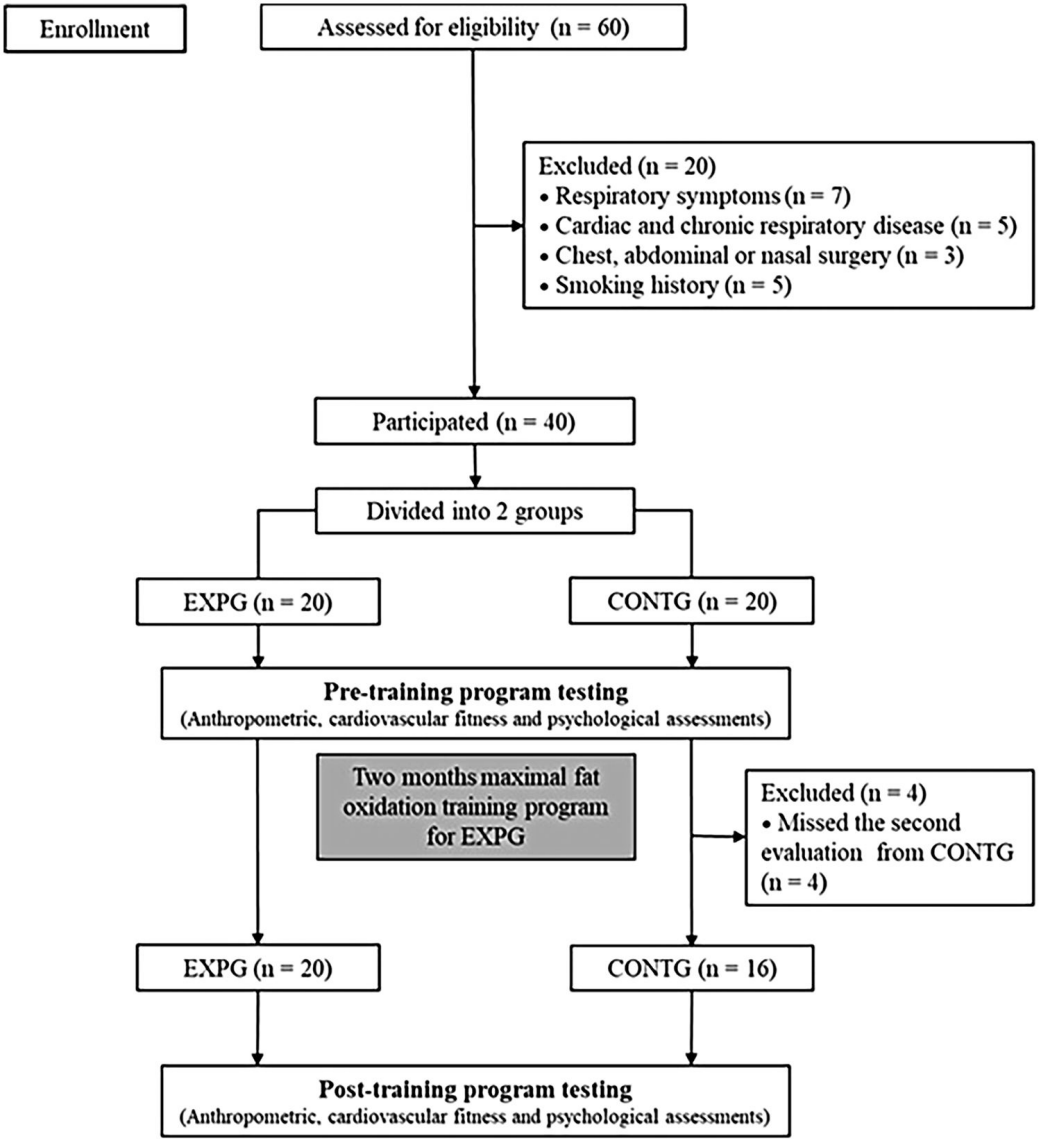
Flow chart of the study.

The outcome measures included anthropometrics [age, height, body mass (BM), body mass index (BMI), waist and hip circumferences (WC and HC), and waist-to-hip ratio (WHR)], cardiopulmonary fitness [relative peak maximal consumption (VO_2_ peak) and relative maximal fat oxidation (FATmax) rate], and psychological questionnaires [anxiety, depression, and self-esteem]. These assessments were conducted in the school setting before and after the two-month training program (Figure 1).

### 2.2 Participants

Thirty-six school-aged children with obesity voluntarily participated in this study (age: 13.1 ± 0.9 years; body mass: 90.6 ± 11.3 kg; height: 1.64 ± 0.07 m; BMI: 33.4 ± 2.3 kg.m^−2^; Pubertal stage: 3.4 ± 1.0 PS). The sample size was calculated according to Faul et al. (19) procedures’ using the G*Power software (version 3.1.9.4; Kiel University, Kiel, Germany). The *α* value was set at 0.05 and the power (1−*β*) at 0.85. Based on the results of Ben Ounis et al. (11), the effect size estimated was >0.8 (Large effect). Fifteen participants per group would provide sufficient power to minimize the risk of committing a Type II statistical error. The participants were selected based on the International Obesity Task Force's inclusion criteria, which required a BMI above the 97th percentile (20). To ensure a diverse and representative sample, participants were recruited from both rural and urban settings, with clinical data collected via a standardized questionnaire (21). The study involved 36 children out of 60 who were eligible for the study intervention. Twenty participants were excluded due to respiratory symptoms (*n* = 7), cardiac disease or acute chronic respiratory diseases (*n* = 3), chest, abdominal, or nasal surgery (*n* = 5), and smoking history (*n* = 5). Participants were randomly assigned to a control group (CONTG, *n* = 16) and an experimental group (EXPG, *n* = 20). Randomization was performed with the ‘‘RAND’' formula in Microsoft Excel, and allocation concealment was maintained until baseline measurements were taken to prevent selection bias. Stratification variables such as age, pubertal stage, and geographical location were not utilized due to a small sample size, despite being documented in baseline measures. Only EXPG participated in the training program. Four children from the CONTG missed the second assessment and were excluded from the analysis (Figure 1). The study involved children and their parents being informed about the risks and benefits of participating in experimental procedures before giving their written informed consent. The study protocol was approved by the University's Institutional Ethical Committee and the Ministry of Education and Higher Education and Scientific Research (MEHESR-2005/014; January 5, 2025), following the Declaration of Helsinki (2013).

### 2.3 Anthropometric assessments

A medical checkup was conducted, and anthropometric measurements were taken from each participant. Body weight was recorded to the nearest 0.1 kg using a digital scale (OHAUS, Florhman Park, NJ), and height was measured to the nearest 0.1 cm using a standing stadiometer. WC and HC were measured using a ribbon meter with a precision of 0.1 cm. The BMI was determined by dividing body mass in kilograms by the square of height in meters, and the WHR was calculated. All measurements were carried out twice by a single competent technician (ISAK Level 1) with an intra-observer technical error of measurement below 1% in all the variables, according to standard protocols. Pubertal status was confirmed for all participants by the school pediatrician at baseline and the two-month follow-up, based on Tanner classification (22) of pubic hair development.

### 2.4 Cardiopulmonary testing

Two progressive cycle ergometer tests were conducted before and after the 2-month intervention. The children had a familiarization session with the cycle ergometer 48 h before the exercise testing. The tests were conducted in a laboratory setting after approximately 12 h of sleep, with a temperature range of 22–24 °C and a relative humidity of 76%. Using an electromagnetically braked cycle ergometer (Ergoline, Bitz, Germany), participants performed the previously reported exercise methodology (23). The test protocol consists of a 5-stage progressive submaximal exercise corresponding to 20, 30, 40, 50, and 60% of Wmaxth, with a 6-minute exercise at each work rate and a 4-minute active recovery period at 20% of Wmaxth. Peak oxygen uptake (VO_2_ peak) and gas exchange (VO_2_ and VCO_2_) were measured via breath-by-breath sampling of expired gases during testing using a metabolic gas analyzer (ZAN 600; ZAN Me*β*geräte, Oberthulba, Germany). The ZAN 600 gas analyzer was calibrated before each test using certified gases (16% O_2_, 4% CO_2_). Day-to-day coefficient of variation for VO_2_ and VCO_2_ was <2%, consistent with published validation data.^1^ VO₂ peak was defined as the highest 30-s average VO₂, with maximal effort confirmed by HR ≥95% of predicted maximum and RER ≥1.00 (24).

Heart rate was monitored continuously throughout the test using ECG (ZAN ECG 800; ZAN Me*β*geräte). Participants’ theoretical maximal oxygen intake (VO_2_maxth) and aerobic workload (Wmaxth) were determined using prediction equations considering sex and anthropometric characteristics (25): VO_2_maxth (mL/min) = [28.5 × body mass (kg)] + 288.1 and the following equation was used to calculate Wmaxth:={VO_2_maxth−10 × [body mass (kg)]}/10.3.

After two months, the children performed the same exercise test protocol at the same incremental workload and were compared at the same percentage of their Wmaxth. Ventilatory parameters were recorded during the last 3 min of each workload to calculate substrate oxidation flow rates (26). Whole-body substrate oxidation was calculated from the respiratory exchange ratio, while fat and carbohydrate oxidation rates were calculated using the non-protein respiratory quotient technique (27), as protein breakdown contributes little to energy metabolism during exercise (28). In this study, we calculated the maximal fat oxidation (FATmax) point to represent the balance between fat and carbohydrate utilization induced by increasing exercise intensity (23). This point reaches maximum fat oxidation induced by workload, followed by a decrease as carbohydrate becomes the predominant fuel, allowing for individualized training.

### 2.5 Anxiety assessment

The study used the French version of Spielberger's State-Trait Anxiety Inventory (STAI-Y), a widely used tool for assessing trait and state anxiety, to diagnose and differentiate anxiety from depressive disorders, with 20 questions for the trait anxiety section (29). State anxiety items include: “I am tense; I am worried” and “I feel calm; I feel secure.” Trait anxiety items include: “I worry too much over something that doesn’t matter” and “I am content; I am a steady person.” All items are rated on a 4-point scale (e.g., from “Almost Never” to “Almost Always”). Higher scores indicate greater anxiety. The STAI is suitable for individuals with at least a sixth-grade reading level.

### 2.6 Depression assessment

The study used the French version of the Beck Depression Inventory (BDI), a 21-item self-reported rating tool used to assess the characteristics and symptoms of depression, with each item scored from 0 to 3. The total score, ranging from 0 to 63, provides a quantitative evaluation of the depressive state. The abbreviated formula categorizes depression scores into no depression (0–4), slightly depressed (5–7), moderately depressed (8–15), and severely depressed (16 and over) (30). The BDI is also suitable for individuals with at least a sixth-grade reading level.

### 2.7 Self-esteem assessment

The study used the French version of Rosenberg's self-esteem questionnaire (31), a widely used self-assessed measure in social science research, with a score below 15 indicating low self-esteem, based on a scale from 0 to 30. The questions consist of 10 Likert-type scale items ranging from 1 (strongly disagree) to 4 (strongly agree), with five items scored positively and five negatively.

### 2.8 FATmax training

Participants completed a physical activity readiness questionnaire, indicating that they only participated in 1 h of school physical education class per week. The CONTG maintained its usual level of physical activity, while the EXPG participated in a supervised exercise training program for two months consisting of 4 weekly sessions of 90 min each, as described by Ben Ounis et al. (11). Each supervised 90-min session consisted of: 10-min warm-up, 60–65 min of moderate aerobic exercise at FATmax heart rate, and 15–20 min of cool-down. Heart rate was continuously monitored to ensure safety for children with obesity (11, 16). They received a two-page summary from an exercise physiologist detailing the health benefits of regular exercise and recommendations and precautions for exercise. The exercise routine included warming up, jogging, jumping, and ball games. The intensity was adjusted based on the participant's heart rate, which was monitored using a heart rate monitor (Vantage NV; Polar Electro, Kempele, Finland). Briefly, the training session schedule included a 10-min warm-up, two 30–35-min blocks of ludic, group-exercise (e.g., relay team races, obstacle courses, modified sport) performed at the correspondent FATmax's heart rate (±10 bpm), 5 min of active recovery, 30-min training at the FATmax's heart rate, and 10 min of cool down. Although FATmax was assessed via cycle ergometry, dynamic field activities (e.g., relay team competition, obstacle course, modified sport) were chosen for the training intervention. Heart rate was maintained within the FATmax zone (±10 bpm) to ensure metabolic consistency. This approach improved compliance and ecological validity in a pediatric population while ensuring that participants maintained the target heart rate associated with FATmax intensity. A detailed session-by-session protocol is provided is the Supplementary Material.

### 2.9 Statistical analyses

Data were presented as mean ± SD. Statistical analyses were performed using the SPSS package (SPSS Inc. Chicago. IL. version 26.0). After conducting the Shapiro–Wilk test for normality, Levene's test for homogeneity of variances, and Mauchly's test for sphericity, an independent *t*-test was performed to assess significant differences in baseline values between groups. Separate mixed-factors ANOVA (time  ×  group) for repeated measurements was used to analyze changes in dependent parameters. The main effect for the group (two levels: EXPG and CONTG) and time (two levels: pre- and post-training) was determined through *post-hoc* analysis with Bonferroni's correction. *post-hoc* analysis using Bonferroni's correction was then performed to calculate the main effect for the group (two levels: EXPG and CONTG) and time (two levels: pre- and post-training). Partial eta-squared (*η*_p_^2^) was quantified for analysis of variance of repeated measures (32). Effect sizes were classified as small (*η*_p_^2^ up to 0.059), medium (between 0.059 and 0.138), and large (greater than 0.138). Between-groups standardized mean differences or effect sizes (ES) were calculated using Cohen's *d* and corrected by Hedge's *g* to avoid biased estimation of the population effect size. ES can be classified as small (0 ≤ *d* ≤ 0.49), medium (0.50 ≤ *d* ≤ 0.79), and large (*d* ≥ 0.80) (33). The level of significance was set at *p* < 0.05.

### 3 Results

The mean values of anthropometric characteristics, self-esteem, anxiety, depression scores, and cardiopulmonary fitness in pre-and post-FATmax training are illustrated in Table 1.

**Table 1:** Effect of FATmax training program on anthropometric, psychological scores and cardiopulmonary fitness.

Variable	CONTG (*n* = 16)		EXPG (*n* = 20)		*η*_p_^2^ (group)/*p*-value	*η*_p_^2^ (time)/*p*-value	*η*_p_^2^ (time x group)/*p*-value
	Pre-intervention	Post-intervention	Pre-intervention	Post-intervention			
Age (year)	13.2 ± 1.0		13.1 ± 0.9				
Pubertal stage (Tanner)	3.4 ± 1.0		3.4 ± 1.0				
Environment (Urban/Rural)	8/8		11/9				
BM (kg)	90.3 ± 11.1	91.7 ± 11.9	90.9 ± 11.4	85.0 ± 10.6	0.02/0.4	0.60/0.000	0.79/0.000
ES	1.4% (−0.12)		−7% (0.5)** ^††^				
BMI (kg.m^−2^)	33.3 ± 2.3	33.8 ± 2.9	33.5 ± 2.3	31.4 ± 2.2	0.08/0.1	0.61/0.000	0.80/0.000
*Δ* (ES)	1.5% (−0.19)		−6.9% (0.93)** ^††^				
WC (cm)	104.1 ± 11.4	104.7 ± 11.3	105.5 ± 10.9	95.7 ± 10.2	0.03/0.28	0.97/0.000	0.98/0.000
*Δ* (ES)	0.6% (−0.05)		−10.3% (0.91)** ^††^				
HC (cm)	111.1 ± 9.5	111.7 ± 9.6	112.1 ± 9.0	103.5 ± 9.1	0.004/0.25	0.87/0.000	0.90/0.000
*Δ* (ES)	0.5% (−0.06)		−8.3% (1.06)** ^††^				
WHR (cm)	0.94 ± 0.07	0.94 ± 0.07	0.94 ± 0.06	0.93 ± 0.06	0.001/0.85	0.12/0.023	0.17/0.012
*Δ* (ES)	0.17% (−0.014)		−1.8% (0.24)				
VO2 peak (mL.kg^−1^.min^−1^)	21.9 ± 3.2	22.0 ± 3.5	21.9 ± 3.1	28.4 ± 4.1	0.22/0.004	0.95/0.000	0.93/0.000
*Δ* (ES)	0.2% (0.03)		23% (1.79)** ^††^				
FATmax rate (mg.kg^−1^.min^−1^)	1.40 ± 0.1	1.39 ± 0.2	1.39 ± 0.2	2.30 ± 0.4	0.90/0.00	0.94/0.000	0.93/0.000
*Δ* (ES)	−0.4% (−0.06)		66% (2.88)*** ^†††^				
Self-esteem (AU)	21.8 ± 4.2	21.4 ± 4.7	23.7 ± 4.9	37.5 ± 6.5	0.44/0.00	0.94/0.000000	0.94/0.000
*Δ* (ES)	−2% (−0.09)		59% (2.35)*** ^†††^				
Anxiety (AU)	30.3 ± 6.5	31.1 ± 6.8	31.3 ± 7.2	27.5 ± 7.7	0.008/0.61	0.58/0.000	0.78/0.000
*Δ* (ES)	3% (−0.12)*		−12% (0.50))** ^††^				
Depression (AU)	14.5 ± 3.9	15.2 ± 4.6	13.4 ± 2.6	9.6 ± 3.0	0.19/0.007	0.68/0.000	0.82/0.000
ES	5% (−0.16)*		−28% (1.33)*** ^†††^				

BM: body mass; BMI: body mass index: WC: waist circumference: HC: hip circumference; WHR: waist-to-hip ratio; VO_2_peak: peak of oxygen consumption; FATmax: maximal fat oxidation; *Δ*: percentage changes; ES: Effect size; within group difference: *p < 0.05, **p < 0.01; between groups difference: ^†^*p* < 0.05, ^††^*p* < 0.01.

### 3.1 Anthropometric data

No significant differences in age, pubertal stage, or geographical location were observed between the EXPG and CONTG at baseline (*p* values ranging from 0.75 to 0.99). EXPG showed significant pre-to-post reductions in all anthropometric indices (all *p* < 0.001), whereas CONTG exhibited small but significant increases in BMI and WHR (*p* < 0.05).

Significant interactions were found (training  ×  group) for BM, BMI, WC, HC, and WHR (F_(1.34)_ = 129.4, *η*p^2^ = 0.79; F_(1.34)_ = 134.9, *η*p^2^ = 0.80; F_(1.34)_ = 372.5, *η*p^2^ = 0.92; F_(1.34)_ = 296.7, *η*p^2^ = 0.90; F_(1.34)_ = 7.1, *η*p^2^ = 0.17, respectively). *post-hoc* analysis revealed that body composition indices (BM, BMI, WC, HC, and WHR) improved only in EXPG (*p* < 005 to *p* < 0.001).

### 3.2 Cardiopulmonary data

EXPG's baseline FATmax rate and VO_2_ peak were 1.4 mg/kg/min and 21.9 mL/kg/min, respectively. After the 2-month FATmax training, these increased to 2.3 mg/kg/min and 28.4 mL/kg/min, ranging from 23% for VO_2_ peak to 66% for FATmax rate. These are significantly higher than CONTG, which showed a 0.2% increase in VO_2_ peak and −0.4% decrease in FATmax rate, respectively.

Significant interactions were also found for FATmax rate and VO_2_ peak (F_(1.34)_ = 470.0, *η*_p_^2^ = 0.93; F_(1.34)_ = 469.3, *η*_p_^2^ = 0.93, respectively). *post-hoc* analysis revealed that the values of the FATmax rate and VO_2_ peak increased in EXPG (*p* < 0.001).

### 3.3 Psychological data

EXPG's baseline self-esteem, anxiety, and depression scores were 23.7, 31.3, and 13.4, respectively. After the 2-month FATmax training, these increased to 37.5 for self-esteem and dropped to 27.5 and 9.6 for anxiety and depression, respectively, ranging from 12% for anxiety to 59% for self-esteem. These are significantly higher than CONTG, which showed a 2% decrease in self-esteem scores and a 3% and 5% increase in anxiety and depression scores, respectively.

Significant interactions were also found for self-esteem, anxiety, and depression scores (F_(1.34)_ = 554.4, *η*_p_^2^ = 0.94; F_(1.34)_ = 120.8, *η*_p_^2^ = 0.78; F_(1.34)_ = 149.8, *η*_p_^2^ = 0.82, respectively). *post-hoc* analysis revealed that the values of the self-esteem score increased, and anxiety and depression scores decreased in EXPG (*p* < 0.01 to *p* < 0.001). Moreover, *post-hoc* analysis revealed that the values of anxiety and depression scores increased in CONTG (*p* < 0.05).

**Figure 2:**
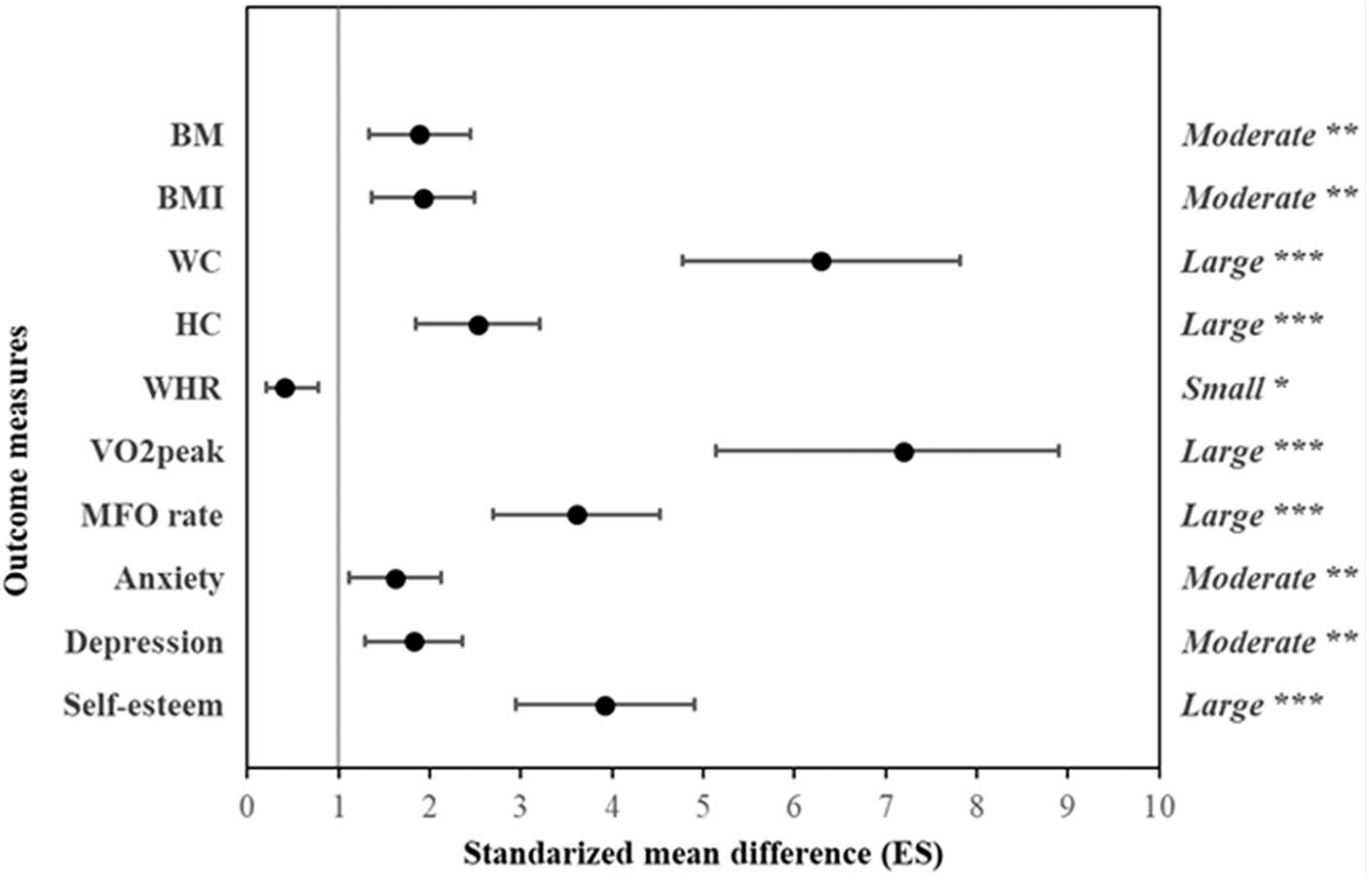
Between-groups comparison for anthropometric characteristics, cardiopulmonary fitness, and psychological questionnaire scores. Bars represent uncertainty in real mean changes with 95% confidence intervals. BM: body mass; BMI: body mass index; WC: waist circumference; HC: hip circumference; WHR: waist-to-hip ratio; VO2peak: peak of oxygen consumption; FATMAX rate: maximal fat oxidation rate. *p < 0.05; **P < 0.01; ***P < 0.001.

Figure 2 displayed the between-group comparison of body composition data, VO_2_ peak, and FATmax rate values as well as anxiety, depression, and self-esteem scores. The improvement in BM [ES: 1.88 (95% CI: 1.34–2.45)], BMI [ES: 1.93 (95% CI: 1.37–2.49)], WC [ES: 6.29 (95% CI: 4.76–7.81)], HC [ES: 2.35 (95% CI: 1.85–3.21)] and WHR [ES: 0.41 (95% CI: 0.21–0.78)] were significantly greater in EXPG compared to CONTG (small-to-large). In addition, the improvements in VO_2_ peak [ES: 3.61 (95% CI: 2.69–4.52)] and FATmax rate [ES: 3.64 (95% CI: 2.70–4.53)] values as well as anxiety [ES: 1.62 (95% CI: 1.12–2.12)], depression [ES: 1.82 (95% CI: 1.28–2.36)], and self-esteem scores (ES = 3.92 [95% CI: 2.94–4.90] were significantly greater in EXPG compared to CONTG (moderate-to-large).

The relationship between changes in psychological scores and changes in anthropometric characteristics and physical fitness is summarized in Table 2. When both groups' data were pooled, there were positive and negative relationships between the percentage of changes in anxiety, depression, and self-esteem scores with the percentage of changes in BM, BMI, WC, HC, and WHR (r = −0.16 to r = 0.84; *p* < 0.05 to *p* < 0.001). In addition, the percentage of changes in anxiety, depression, and self-esteem scores also showed positive and negative relationships with the percentage of changes in VO_2_ peak and FATmax rate values (r = −0.72 to r = 0.91; *p* < 0.01 to *p* < 0.001). The percentage of change in VO2 peak showed a positive relationship with the percentage of changes in Fatmax rate (r = 0.94; *p* < 0.001).

**Table 2:** Correlation analysis between changes in psychological scores and changes in body composition and cardiopulmonary fitness indices.

Variable	Self-esteem	Anxiety	Depression
BM	−0.73**	0.63**	0.70**
BMI	−0.82***	0.58**	0.64**
WC	−0.89***	0.74**	0.84***
HC	−0.87***	0.71**	0.72**
WHR	−0.16*	0.18*	0.27*
FATmax rate	0.86***	−0.81***	−0.82***
VO_2_ peak	0.91***	−0.72**	−0.74**

BMI, body mass index; WC, waist circumference; HC, hip circumference; WHR, waist-to-hip ratio; VO_2_peak, peak of oxygen consumption; FATmax, maximal fat oxidation; *p < 0.05; **p < 0.01; ***p < 0.001.

## 4 Discussion

The present study aimed to investigate the effect of a two-month maximal fat oxidation (FATmax) training program on psychological well-being and mental health in school-aged children with obesity. The findings of the current study indicate that individualized FATmax training improves metabolic and fitness metrics, as well as psychological well-being, in pediatric individuals with obesity, who are at a high risk for mental health disorders.

The most salient result of the current study is the significant improvement in psychological and well-being outcomes in EXPG. The results of anxiety, depression, and self-esteem scores showed a significant interaction effect (group  ×  time) with a moderate-to-large effect size in the EXPG group (ES: 1.62 for anxiety, 1.82 for depression, and 3.92 for self-esteem). The literature on the impact of training programs on mental health in children and adolescents with obesity is scarce and presents conflicting results (8, 16). Yu et al. (8) showed that school-based nutrition education and physical activity intervention improved well-being and social anxiety scores in 99 children with obesity but did not alter depression scores. Conversely, Migueles et al. (16) found no significant mental health effects of 20 weeks of aerobic plus resistance exercise training on 92 overweight or children with obesity. This inconsistency can be attributed to factors including exercise training type, duration, frequency, session adherence, participant characteristics, and baseline psychological and mental health status. Potentially, the training settings applied in the current study, i.e., FATmax intensity, 4 sessions a week, each lasting 90 min for EXPG, were barely adequate for improving psychological and mental health outcomes (7). There is not only one mechanism underpinning the significant training impact on EXPG's psychological and mental health but rather a holistic and wide range of factors. Amongst them the effect of exercise on brain activities and biomarkers. Jemni et al. (34) showed that moderate to high-intensity exercise increases brain-derived neurotrophic factor (BDNF) levels in both healthy individuals and clinical populations, subsequently stimulating serotonin production. The latter is considered the hormone of joy and plays a significant role in mental well-being. Exercise-related Lactate can cross the blood-brain barrier via specific transporters. The transportation of lactate across the blood-brain barrier may be the necessary link correlating how physical exercise is directly involved in the BDNF-dependent neurobiological pathways; hence, the more lactate is produced, the more it crosses the blood-brain barrier, and the more BDNF is secreted in the bloodstream (34). High-intensity exercise can elevate BDNF through lactate-dependent mechanisms, but specialized FATmax intensity training results in minimal lactate production. Improvements in self-esteem and reduced anxiety are more likely mediated by enhanced cerebral blood flow, direct BDNF stimulation, and improved body image satisfaction resulting from reduction in central adiposity (34, 35).

The results of the body composition showed a significant (group  ×  time) interaction effect for BM, BMI, WC, HC, and WHR, with a small to substantial effect size observed in the EXPG (ES: 1.88 for BM, 1.93 for BMI, 6.29 for WC, 2.53 for HC, and 0.41 for WHR). These findings are consistent with previous studies using moderate aerobic exercise training programs in diverse groups of individuals with obesity (10, 11, 14). Tan et al. (13) found that 10 weeks of FATmax training significantly reduced body mass (−1.5 kg), BMI (−1.2 kg/m^2^), fat mass (−1.2 kg), and abdominal fat (−0.13 kg) in boys with obesity. Furthermore, Huang et al. (10) concluded that moderate-intensity combined exercise was the best mode for weight loss. The study's body composition improvement may be attributed to increased energy requirements from the exercise training program, which activated muscle mass, leading to higher energy expenditure via stimulation of fat oxidation, as suggested by previous research (9, 15).

The stimulation of fat oxidation is a result of enhancements in cardiorespiratory fitness. Indeed, the results of cardiopulmonary fitness showed a significant (group  ×  time) interaction effect for both VO_2_ peak and FATmax rate, with a substantial effect size observed in the EXPG (ES: 3.61 for VO_2_ peak and ES: 3.64 for FATmax rate). These results corroborate those of several previous findings (9, 11, 13). Ben Ounis et al. (11) found a 23% increase in VO_2_ peak and a 54% increase in FATmax rate in children with obesity following a similar FATmax exercise-training program. In contrast, the study by Tan et al. (13) showed that boys with obesity who underwent 10 weeks of FATmax training showed an increased FATmax rate from 0.35 ± 0.12 g/min to 0.38 ± 0.13 g/min, but this change was not statistically significant. The discrepancies in these results could be explained by exercise training modalities (cycling vs. running) and duration, age, and gender of participants, as well as the training session's volume. The increase in the FATmax rate observed in the EXPG could be attributed to exercise training, which enhances muscle mass, boosts metabolic rate, and aids in fat burning. It enhances insulin sensitivity in children with obesity, boosting glucose use and lipid breakdown (9). Additionally, it improves mitochondrial density and activity in muscle cells (15).

The training strategy focuses on increasing energy expenditure and fat burning, leading to improved cardiopulmonary fitness and body appearance (8, 10). Self-esteem, body image, and stressful events are considered the most reliable predictors of anxiety and depression in adolescents (36). Specifically, the EXPG children's positive perception of their physical appearances after training resulted in increased self-esteem and decreased depression and anxiety scores, supported by correlations between changes in anxiety, depression, self-esteem scores, and body composition, especially WC and HC, and cardiopulmonary fitness. (r = −0.63 to r = 0.91). In contrast, CONTG children reported significant increases in anxiety and depression scores. In addition, the improvement in mental health and well-being in EXPG could be attributed to ‘‘the floor effect’’, i.e., most of the children had unsound health at baseline. Lifestyle factors, including a healthy diet, greater physical activity, smoking cessation, and avoidance of alcohol and illicit substances, play an important role in positively modifying medical and psychiatric diseases and their associated morbidity and mortality (37). Other lifestyle factors include a safe environment, optimal sleep, enjoyable activities, social connections/support, and healthy mental activities (37). The study's EXPG may have benefited from the positive factors mentioned, as they have been informed about the health benefits of regular exercise. The positive psychological responses observed after the exercise training intervention suggest an adaptive response to the FATmax training program, possibly due to physiological mediators like cortisol, dopamine, serotonin, and brain-derived neurotrophic factors (11, 15, 34).

### 4.1 Study limitations

The study has identified some limitations that need to be addressed. First, participants self-reported their mental health status using validated French versions of STAI-Y and BDI, potentially causing memory and response bias. Future research should consider incorporating age-specific or observer-rated psychological instruments. Despite this, standardized questionnaires for measuring children's mental health have achieved satisfactory results in previous epidemiological studies. Second, the lack of dietary control among participants may confound results since they maintained habitual diets, but unmeasured nutritional changes could still occur. Third, this study did not assess physiological and biological mediators like hormonal and inflammatory markers, which could explain psychological adaptation processes from FATmax exercise training. In addition, the randomization procedure did not include stratification by relevant baseline variables such as age, pubertal stage, or geographical location (rural/urban), which could affect group comparability. Finally, the transfer of FATmax from cycling to games may reduce exercise specificity.

## 5 Conclusions

This study investigated the effect of a two-month individualized FATmax program on mental health in school-aged children with obesity. It found that the program improved mental health, increased self-esteem, reduced anxiety and depression, improved body composition, and enhanced cardiopulmonary fitness. The research suggests that FATmax exercise training could help children manage their psychological conditions and prevent complications. Further research is needed to understand the physiological mechanisms behind this positive psychological adaptation.

## Data Availability

The original contributions presented in the study are included in the article/Supplementary Material, further inquiries can be directed to the corresponding author.

## Footnotes

^1^Day-to-day coefficient of variation for VO_2_ and VCO_2_ measurements was <2%, indicating excellent test-retest reliability of the metabolic gas analyzer (ZAN 600).

## Supplementary material

Supplementary Table S1 Detailed session-by-session FATmax training protocol.
