# Supplementary material for: Maximal fat oxidation training improves mental health in children with obesity: a 2-month randomized controlled trial
Source: Front Sports Act Living. 2026 Feb 25;8:1759324. doi: 10.3389/fspor.2026.1759324 (PMC12977972; doi:10.3389/fspor.2026.1759324)
Supplement: Supplementary Table S1 — Detailed session-by-session FATmax training protocol. [file Table1.docx]

**Session Template (Repeated Weekly with Rotating Activities)**

| **Phase** | **Duration** | **Intensity (HR)** | **Activities** |
| --- | --- | --- | --- |
| Warm-up | 10 min | 50–60% HRmax | Light jogging, dynamic stretching, mobility games (e.g., “Simon Says” movements) |
| Block 1 | 30–35 min | FATmax HR ±10 bpm | Ludic aerobic games": relay races |
| Active Recovery | 5 min | <50% HRmax | Walking, slow-paced coordination games (e.g., “Red Light, Green Light”) |
| Block 2 | 30 min | FATmax HR ±10 bpm | Team-based dynamic activities": capture-the-flag |
| Cool-down | 10 min | <50% HRmax | Static stretching, breathing exercises, group reflection on effort and enjoyment |

**Weekly Progression & Activity Rotation**

| **Week** | **Objective** | **Activities** |
| --- | --- | --- |
| 1–2 | Familiarization | Simple relays, basic obstacle courses, cooperative games (e.g., “Tunnel Ball”) |
| 3–4 | Coordination + Endurance | Agility ladders, shuttle runs with ball handling, team challenges with time goals |
| 5–6 | Team Strategy + Fun | Modified sports (e.g., “Soccer Golf”, “Basketball Knockout”), music-based movement |
| 7–8 | Autonomy & Leadership | Children co-design mini-games; peer-led warm-ups; “Fitness Olympics” with scoring |
